# A Case Report and Literature Review of Icterus Marginatus: Demarcated Subcutaneous Jaundice, A Sign of Retroperitoneal Bile Leak After Laparoscopic Cholecystectomy

**DOI:** 10.7759/cureus.27543

**Published:** 2022-07-31

**Authors:** Jowhara Al Qahtani, Waleed Mahmoud, Haya Al Kuwari, Syed Muhammad Ali

**Affiliations:** 1 Acute Care Surgery, Hamad General Hospital, Doha, QAT; 2 General Surgery, Hamad General Hospital, Doha, QAT; 3 Surgery, Weill Cornell Medicine - Qatar, Doha, QAT

**Keywords:** surgical case reports, laparoscopic cholecystectomy, bile leak, retroperitoneum, icterus marginatus

## Abstract

Laparoscopic cholecystectomy (LC) is one of the most commonly performed general surgical procedures worldwide. Common bile duct (CBD) injuries are occasionally seen after this procedure and manifest as biliary peritonitis or bile collection; however, retroperitoneal bile leak is an extremely rare phenomenon manifesting as yellow discoloration of the abdominal wall, and a few cases are reported in the English literature. In this article, we describe one case of retroperitoneal bile leak that manifested as flank discoloration and its management.

## Introduction

Bile duct injuries (BDI) has slightly higher rates in laparoscopic cholecystectomy (LC) (0.5%-1.4%) when compared to open (0.2%-0.3%) [1-4]. Although the rate after laparoscopic procedures has been decreasing recently, the severity of injuries is increasing. Most BDI is recognized either during the procedure or in the immediate postoperative period, manifesting as bile leak or biliary tract obstruction [3]. Bile can leak intraperitoneally or occasionally retroperitoneal, which is extremely rare [3]. The retroperitoneal space is bounded by the posterior parietal peritoneum and transversalis fascia. Most retroperitoneal leaks present as collection, causing a mass effect, and are considered a source of infection and the result when gall bladder dissection is difficult in cases of the empyema gall bladder, Calot's dissection, or extensive adhesions. It will dissect the investing peritoneum and the best way to avoid excessive traction on the cystic duct [4]. Here, we report a patient who had a retroperitoneal bile leak after LC that manifested as localized skin discoloration in the flank.

## Case presentation

A 40-year-old female presented to the emergency department with upper abdominal pain and vomiting for three days. The pain was located in the right upper quadrant of the abdomen, not radiating to the back and not related to food intake. The patient had no fever, jaundice, or any other associated symptoms. She was vitally stable, and the abdomen was soft and not distended, with tenderness at the right upper quadrant and a negative Murphy’s sign. Her labs showed deranged liver function and increased bilirubin (bilirubin: 49 umol/L, alkaline phosphatase/ALP: 264U/L, aspartate aminotransferase/AST: 266 U/L, and alanine transaminase/ALT: 243 U/L). Her ultrasound (US) of the abdomen showed distention of the gallbladder with multiple calculi. The largest calculus measures 5.7 mm at the neck region with normal gallbladder wall thickening (2.3 mm) and no pericholecystic fluid. CBD measured 6.5 mm, with no filling defect. On the following day, her magnetic resonance cholangiopancreatography (MRCP) showed a CBD filling defect, mostly representing a stone, with dilated proximal CBD measuring 9 mm and dilated intrahepatic bile ducts. The decision was made to proceed with LC with an intraoperative cholangiogram (IOC) and to possibly retrieve the CBD stone.

Intraoperatively, an inflamed gall bladder was found with two proximal cystic duct stones that were removed. Initial IOC showed impacted stone at the distal CBD. Multiple removal trails were attempted using the Dormia basket but failed to clear. Eventually, repeated IOC was done, showing no filling defect in contrast to the duodenum. It was assumed that the stone was pushed to the duodenum. The surgery was concluded smoothly, and the patient was shifted to the recovery room.

Two days postoperatively, the patient complained of persistent diaphoresis, pain in the right upper quadrant, and repeated vomiting episodes. Itching in her groin was associated with light yellow discoloration limited to her pubic area. She had no jaundice or scleral icterus. Her vitals were normal. Blood workups were repeated and showed rising bilirubin levels between 41 and 51 umol/L and increased alkaline phosphatase with mildly deranged liver functions (ALP: 209-224 U/L, AST: 75-25 U/L, and ALT: 170-69 U/L).

**Figure 1:**
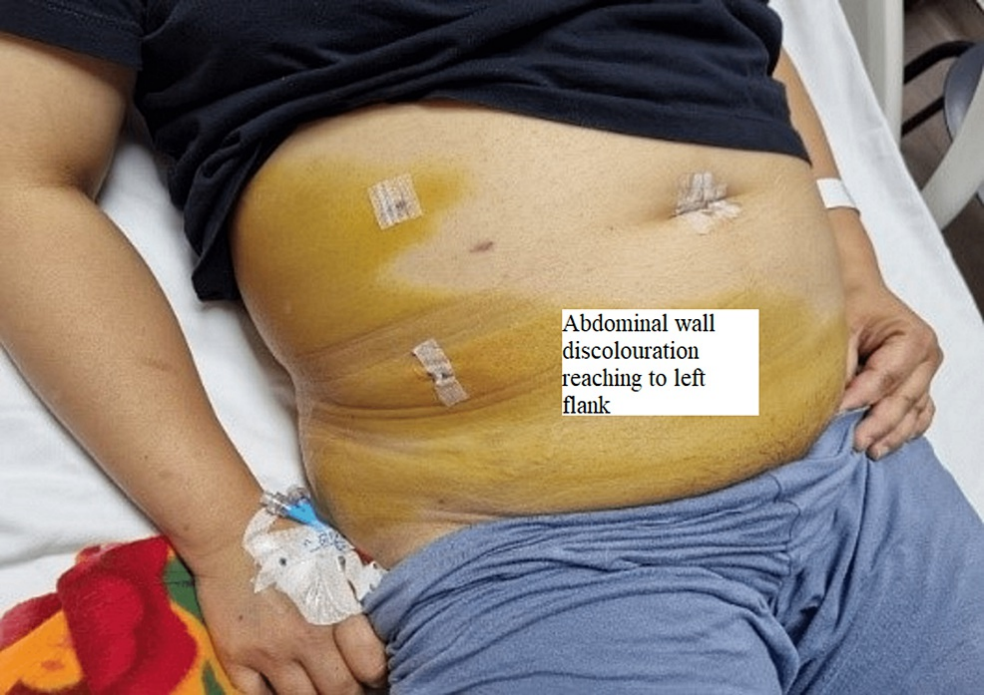
Demarcated line of abdominal wall discoloration

**Figure 2:**
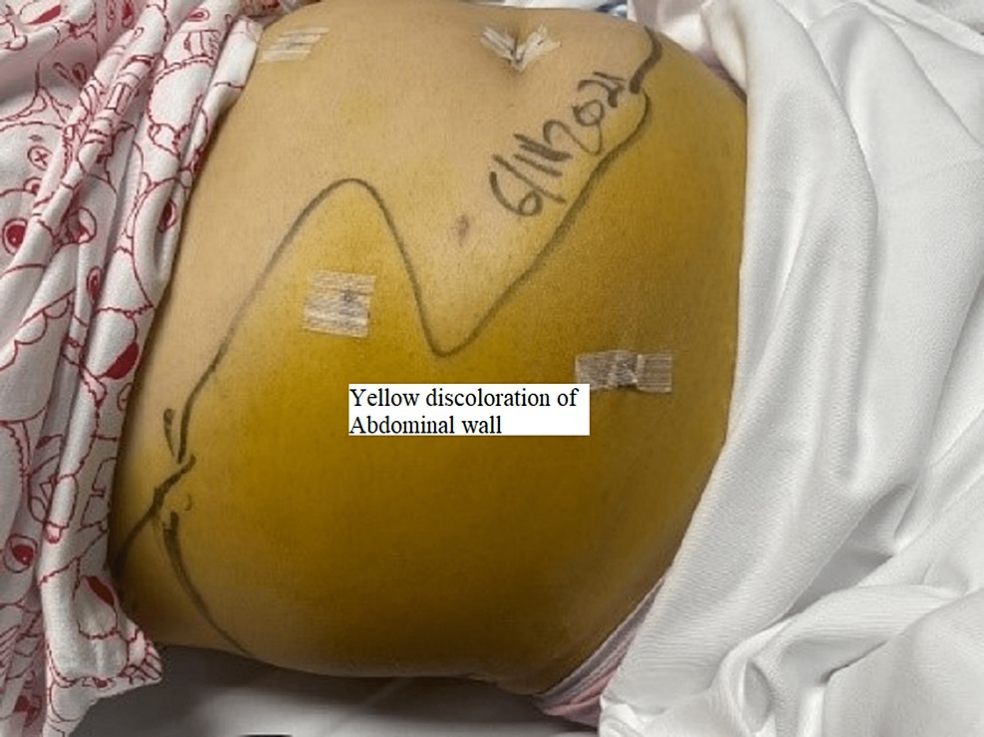
The torso of the patient with yellow demarcated discoloration at the right flank that extends below the right costal margin down to the genitalia and right upper lateral thigh

The patient was started on intravenous fluids, antibiotics, and analgesia. However, her symptoms persisted, and she could not pass gas or stool. Her vitals were unremarkable on the fifth post-surgery day except for tachycardia to 100 bpm. Her physical examination showed a large yellowish discoloration extending from the right flank down to the lower abdomen and the right labia down to the mid-right thigh (Figures 1, 2); it was warm to touch and tender on palpation. Her liver function test (LFT) panel was trending down; however, her CRP (C-reactive protein) went up.

**Figure 3:**
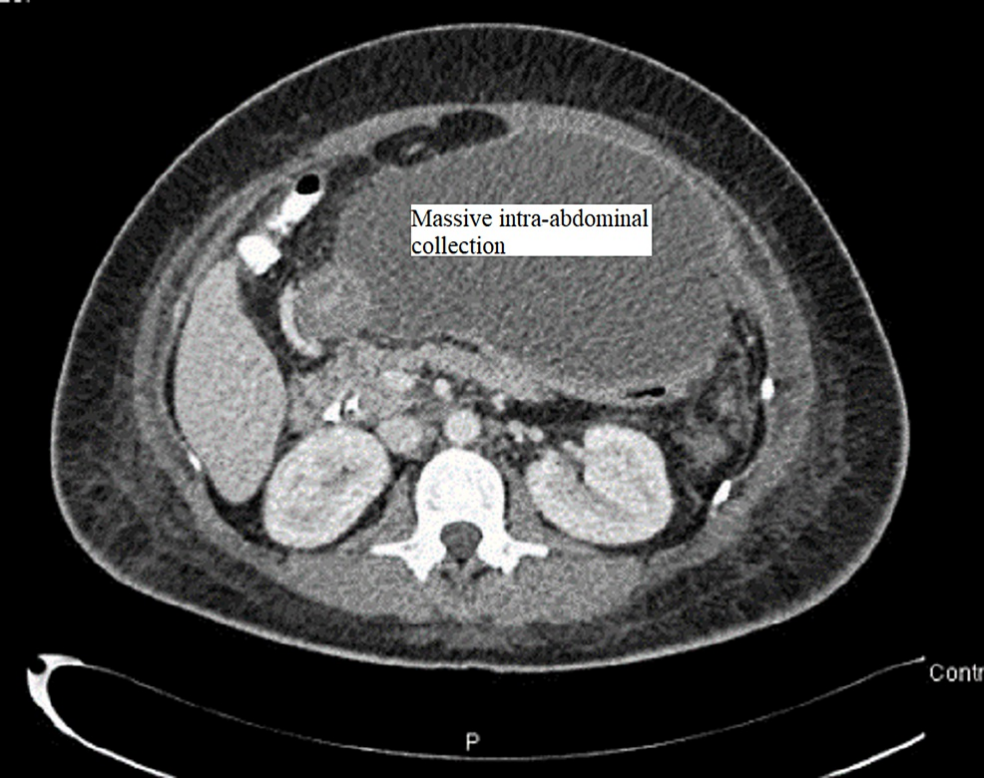
CT abdomen and pelvis with contrast showing the free fluid collection in the upper abdomen

**Figure 4:**
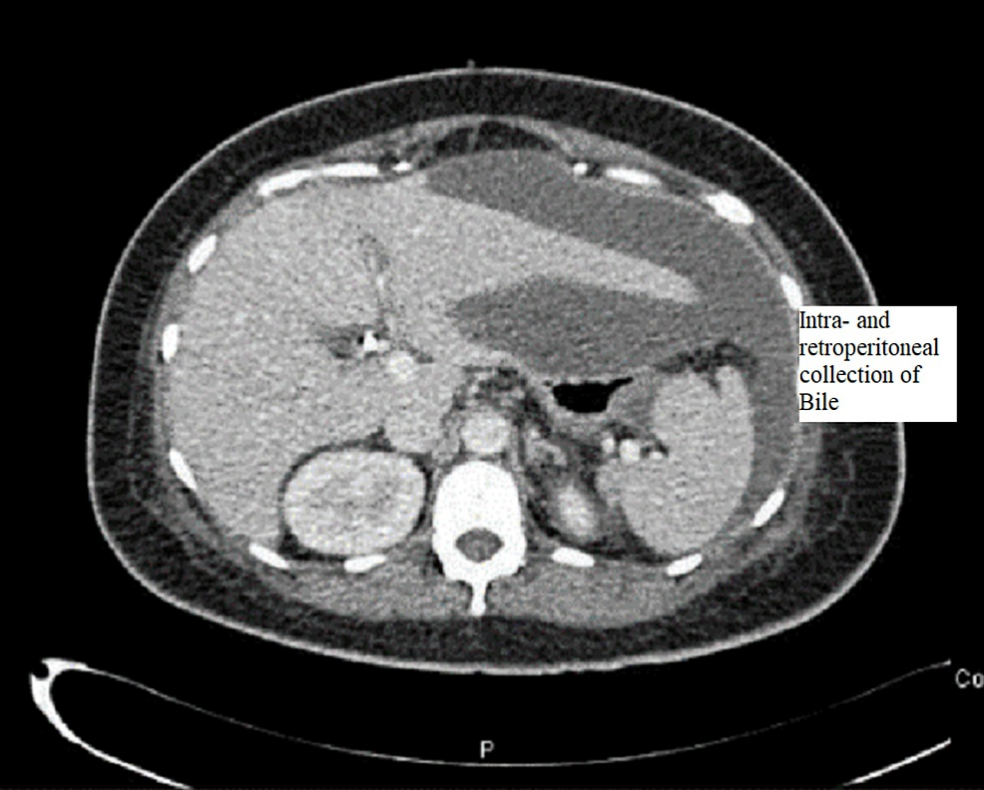
CT abdomen and pelvis with contrast showing the free fluid collection in peri-hepatic and peri-splenic areas

**Figure 5:**
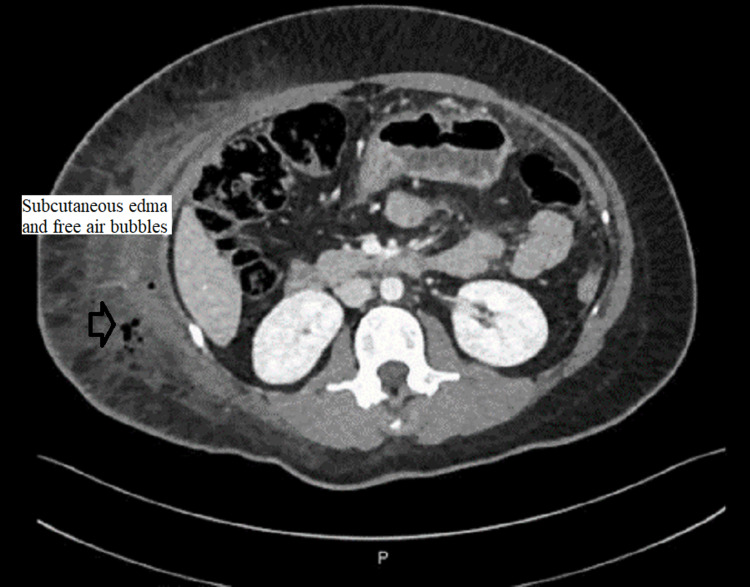
CT abdomen showing extensive edema, fat stranding, and inflammatory changes seen at the right lateral abdominal wall with free air in the subcutaneous soft tissue and muscular planes

CT abdomen was done and showed a large amount of free fluid in the perihepatic, peri-splenic, and pelvic regions, along with extensive subcutaneous edema and stranding with free air within the right lateral abdominal wall (Figures 3-5). In addition, the CT revealed a mild intrahepatic biliary duct and CBD dilatation.

The next day, there was increasing yellowish pigmentation at the abdomen and pelvis, and she became more tachycardic (heart rate 105/minute), yet no fever was documented. Laboratory findings showed increased inflammatory markers (WBC and CRP) with normal lactate. LFT panel was normal except for ALP, which was in the 300s, and total bilirubin was in the 30s.

MRCP was done postoperatively to assess any possible retained stones according to the CBD dilatation previous CT scan despite the clear IOC. MRCP showed a filling defect in the distal end of the CBD with a diameter of 9 mm with a picture of subcutaneous inflammation.

**Figure 6:**
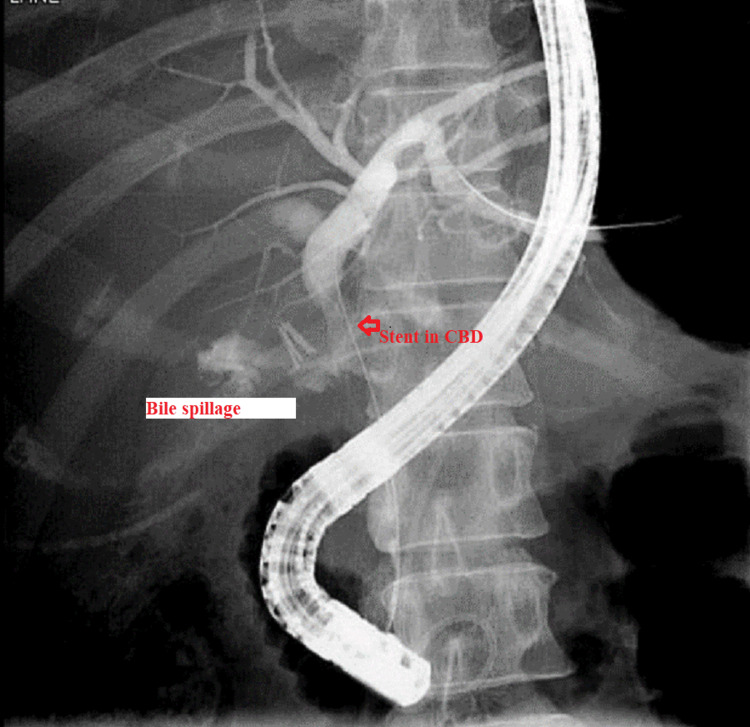
ERCP showing a cystic duct bile leakage after cholangiography ERCP: Endoscopic retrograde cholangiopancreatography; CBD: Common bile duct.

**Figure 7:**
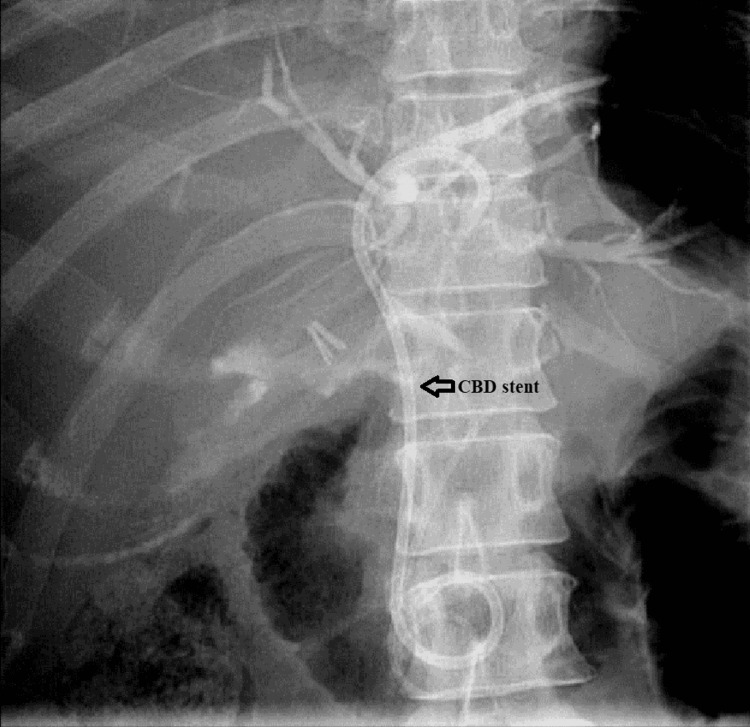
ERCP: 8.5F 5-cm double pigtail plastic biliary stent was placed into the left hepatic duct with good contrast drainage in the duodenum ERCP: Endoscopic retrograde cholangiopancreatography; CBD: Common bile duct.

The patient was maintained on supportive treatment and close observation and was planned for endoscopic retrograde cholangiopancreatography (ERCP) and stenting. It showed a filling defect at the most distal part of CBD. Deep cannulation of CBD was achieved. A contrast leak was detected from the cystic stump with normal intra- and extrahepatic bile ducts. A partial sphincterotomy was performed, and a double pigtail stent was placed at the left hepatic duct, which eventually showed good contrast and bile drainage into the duodenum documented endoscopically and fluoroscopically (Figures 6, 7).

The patient bilirubin level started to trend down, reaching the normal level; however, her inflammatory markers were trending up (WCC 12.7/mm^3^ and CRP 78 mg/L). US of the abdomen showed a large collection at the upper abdomen, and image-guided drainage of the collection was performed by placing an intra-abdominal pigtail drain. Body fluid cultures were sent from the drain and grew *Citrobacter freundii* and *Candida krusei*, which were sensitive to ertapenem. The drain was kept for two weeks for draining the yellow clear fluid.

**Figure 8:**
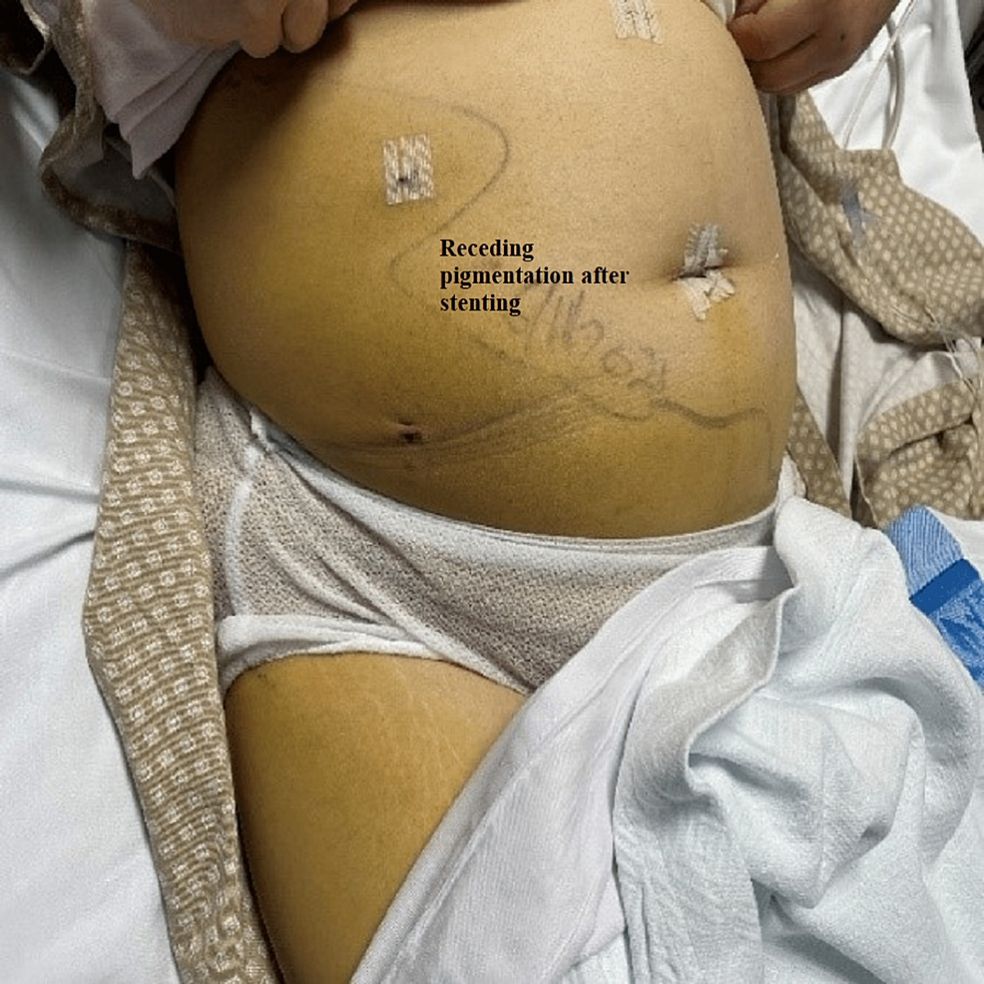
Vanishing demarcated edges of jaundice after ERCP and stenting ERCP: Endoscopic retrograde cholangiopancreatography.

**Figure 9:**
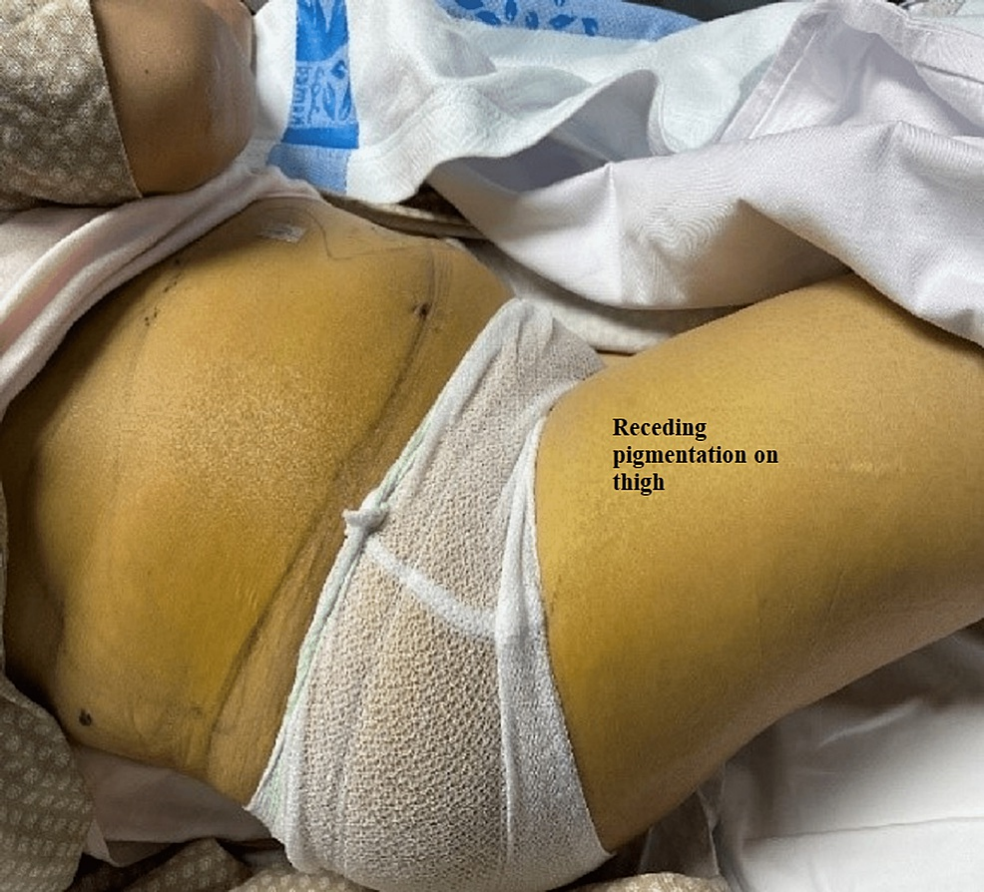
Vanishing demarcated edges of jaundice after ERCP and stenting (lateral view) ERCP: Endoscopic retrograde cholangiopancreatography.

Her condition improved, and abdominal discoloration was fading with decreased demarcation (Figures 8, 9). Her labs kept tending to the normal level (WCC 7.4/mm^3^ and CRP 2.9 mg/L); the pigtail drain was removed, and the patient was discharged three weeks post-surgery in stable condition.

## Discussion

Literature research using PubMed, Google Scholar, and UpToDate was done using all relevant English language studies. The research terms used were "Icterus Marginatus," “retroperitoneal bile leak,” “ CBD (Common Bile Duct) injury and biloma,” “CBD and skin pigmentation,” and “abdominal wall discoloration and jaundice.” Unfortunately, the term "Icterus Marginatus" was only reported in one article by McAlister and Sener [5] and a website [6] with no further details.

The most common fluid collected retroperitoneally is the blood that could manifest subcutaneously in the periumbilical and is called the Cullen sign, which usually indicates severe hemorrhagic pancreatitis [7]. Grey Turner’s sign was associated with ecchymotic discoloration of the flank area and was first seen in 1912 and 1917 and reported in 1919 (Grey Turner). Those signs were associated with severe hemorrhagic pancreatitis, poor outcomes, and high mortality. Additional signs of retroperitoneal bleeding are Bryant’s sign (scrotal ecchymosis), Fox’s sign (upper outer thigh), and Stabler’s sign (pubic and groin bruising) [5].

Retroperitoneal bile leak is rare and primarily due to rupture of the biliary tree. Still, occasionally, it could be secondary to a duodenal perforation due to blunt abdominal trauma, peptic ulcer disease, large impacted stone causing pressure necrosis of the wall, or post-endoscopic procedure such as sphincterotomy [8].

Our patient had a retroperitoneal bile leak shown as a demarcated skin discoloration after LC and IOC. The discoloration was limited to the flank, groin, and genital area. Retroperitoneal bile leak is an infrequent complication as most leaks occur intraperitoneally and present as biliary peritonitis or collection. Few studies reported retroperitoneal bile collection causing sepsis or mass effect, yet those leaks had almost no skin manifestation.

The first document of this demarcated skin discoloration was by Ranshoff in 1906 [9]. He described marked jaundice of the umbilicus of a patient who was admitted with a probable diagnosis of peritonitis of appendiceal origin: “The navel was of a distinct saffron-yellow color in strong contrast with the rest of the skin over the abdomen” [9]. Intraoperatively, it was found that the patient had a bile leak from the posterior wall of the common bile duct (CBD) in the supraduodenal part. It manifested first in the navel because this part is thinner than the rest of the abdominal wall. The bile tracking from the retroperitoneum along the fascial planes is formed by the embryological facial planes [5,10-13].

The literature review found eight studies, including the current case with nine patients who reported similar skin discoloration. Details of these case reports are in Table 1.

**Table 1:** Studies reporting demarcated skin discoloration due to retroperitoneal bile leak ERCP: Endoscopic retrograde cholangiopancreatography; CBD: Common bile duct.

Author	# of cases	Age	M/F	Initial diagnosis	Diagnostic workup/Intervention	Skin color distribution	Days to presentation	Intervention	Finding	Intervention	Outcome
McAlister and Sener 2005 [5]	2	(A) 43	M	Not mentioned	Both had cholecystectomy.	(A) Limited to the flanks and groin bilaterally, more marked on the right side than the left.	(A) Not mentioned	ERCP	Both had a cystic duct bile leak.	Sphincterotomy	Home
		(B) 59	M			(B) Torso, perineum, and upper thighs with a horizontal border in the upper chest and upper thigh.	(B) 5 days				
Ranshoff 1906 [9]	1	53	M	Peritonitis of appendiceal origin	None	Umbilicus	3 days	Laparotomy	Spontaneous supraduodenal, posterior CBD wall perforation	Irrigation, drain placed	Home
Brady et al., 2006 [10]	1	73	M	Strangulated right inguinal hernia	CT scan: mesenteric fact containing hernia with a fluid collection extending along with the iliopsoas muscle and stone in the gallbladder	Right scrotum and right inguinal area	NA	Right inguinal hernia exploration	Mesenteric fact and copious amount of green fluid	CT scanà 1.2 cm calculi within CBD.	Home
										Laparotomy: no perforation or leak or collection. A Fogarty catheter was used to retrieve 2 large stones, and a T-tube insertion was done.	
Neoptolemos et al., 1984 [12]	1	72	M	Choledocholithiasis	ERCP and sphincterotomy	Right flank and scrotum	2 days	Laparotomy	Bile was extending down the right flank into the pelvis along the facial planes into the scrotum. No free bile in the pelvis. The site of perforation was not identified.	Cholecystectomy and kocherization of duodenum choledochotomy, T-tube was inserted in CBD, and drain was placed in the pelvis.	Death (due to bronchopneumonia)
Shahedi and Tejaswi, 2016 [14]	1	25	F	Not mentioned	Laparoscopic cholecystectomy	On abdomen	2 days post-op	CT followed by drainage, then ERCP	Leak at the cystic stump next to surgical clips.	Sphincterotomy and plastic stent placement	Home
Allegue et al., 2009[15]	1	70	F	Choledocholithiasis	ERCP	Yellowish macules on her loins, more intense on the right side	5 days	Initial CT scan and follow-up CT	Initial CT: retroperitoneal fluid, mostly in the anterior and right posterior pararenal spaces. Follow-up CT: gas in the area adjacent to the papilla of Vater (post-ERCP duodenal perforation).	Surgery: duodenal diversion and T-tube insertion	Home
												Fisken et al., 2011[16]	1	Elder patient	M	Pneumonia/acute cholecystitis	Diagnostic laparoscopy	Umbilicus	Few days	Diagnostic laparoscopy	Perforated gangrenous gallbladder with biliary peritonitis.	Subtotal cholecystectomy	Not mentioned
																								This study	1	40	F	Choledocholithiasis	Laparoscopic cholecystectomy and intraoperative cholangiogram	Pubis, right groin, thigh, and flank	1 day	ERCP	A filling defect at the most distal part of CBD. There was a contrast leak from the cystic stump.	A partial sphincterotomy was performed, and a double pigtail stent was placed at the left hepatic duct.	Home

There were six males and three females. In four cases, out of nine, “discoloration” was due to bile leakage from the cystic stump after cholecystectomy, and in two cases, it was due to the perforation of the biliary tree post ERCP and sphincterotomy. One patient had a spontaneous perforation of posterior CBD, another was due to gallbladder perforation, and the last case had no apparent reason for the skin discoloration as no source was identified, i.e., no leak, perforation, or fistula communicating to the peritoneum. This agrees with the literature that most causes of bile leak are iatrogenic, and the most common site is the cystic stump [11-13]. Most patients had skin discoloration localized to the right flank and groin and were seen postoperatively within two to five days. Four patients initially had choledocholithiasis, and five had surgical intervention, while four of them underwent minimally invasive procedures like ERCP and stent placement. Despite the significant intra-abdominal pathology of this skin demarcation, all patients had good outcomes and went home except for one who passed away due to pulmonary issues. In one case, the patient's outcome was not mentioned [13-16].

Management of bile leak surgically carries within it 30% mortality [12]. Management of intraperitoneal bile leak differs from retroperitoneal bile leak. Therefore, one should have a high clinical suspicion of retroperitoneal leak upon finding icterus marginatus. Lim et al. proposed an algorithm to help surgical teams identify future retroperitoneal bile leaks [13]. Figure 5 shows the algorithm for CBD leaks.

**Figure 10:**
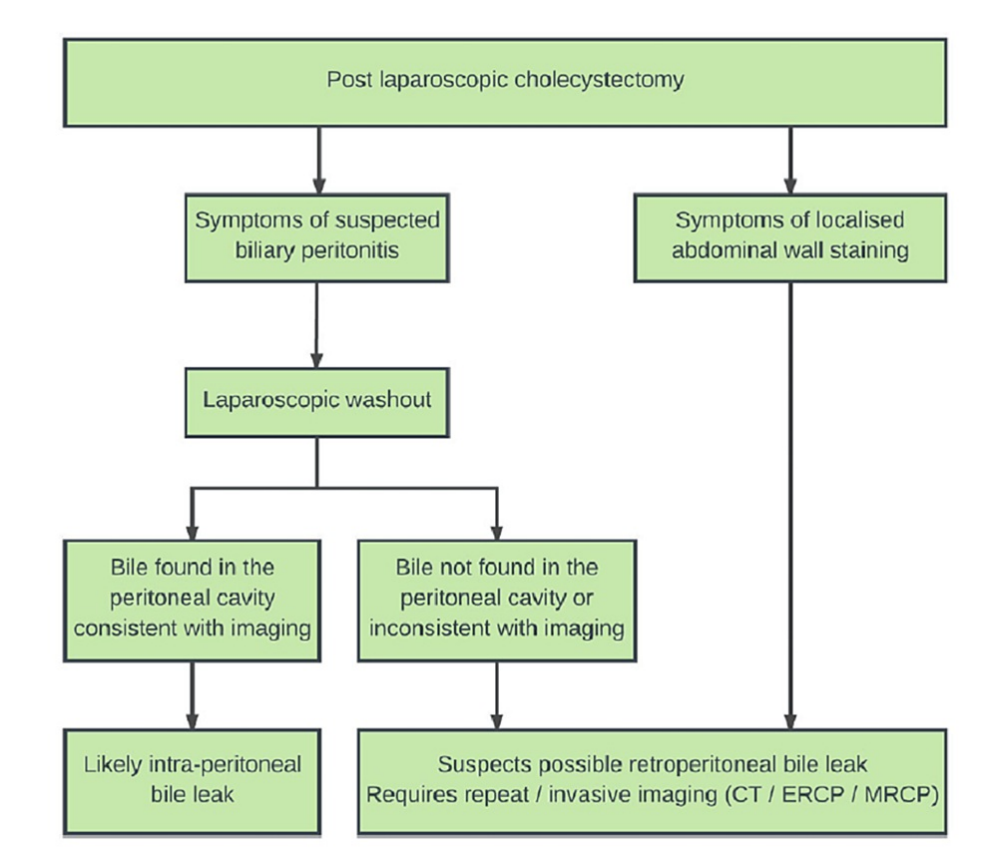
Algorithm to manage retroperitoneal bile leak ERCP: Endoscopic retrograde cholangiopancreatography; MRCP: Magnetic resonance cholangiopancreatography.

## Conclusions

Retroperitoneal bile leak is extremely rare as only a few cases are reported, and it results from an inadvertent breach in the posterior peritoneum during LC and bile leak from the CBD injury. Diagnosis involves the high clinical suspicion and unmistakable typical yellow discoloration of the flank and anterior abdominal wall along with US and MRCP. Endoscopic stenting usually treats the condition depending upon the site and size of the leak. Operative management may be necessary if endoscopic stenting fails. The best way to avoid this complication is careful dissection in the Calot's triangle and minimizing the traction on the cystic duct to keep the retroperitoneum intact.
